# Ethical conflicts in patient relationships: Experiences of ambulance nursing students

**DOI:** 10.1177/0969733020911077

**Published:** 2020-04-07

**Authors:** Anders Bremer, Mats Holmberg

**Affiliations:** Linnaeus University, Sweden; Region Kalmar County, Sweden; University of Borås, Sweden; Linnaeus University, Sweden; Uppsala University, Sweden; Region Sörmland, Sweden

**Keywords:** Ambulance service, clinical studies, ethical conflicts, nursing students, patient relationship, thematic analysis

## Abstract

**Background:**

Working as an ambulance nurse involves facing ethically problematic situations with multi-dimensional suffering, requiring the ability to create a trustful relationship. This entails a need to be clinically trained in order to identify ethical conflicts.

**Aim:**

To describe ethical conflicts in patient relationships as experienced by ambulance nursing students during clinical studies.

**Research design:**

An exploratory and interpretative design was used to inductively analyse textual data from examinations in clinical placement courses.

**Participants:**

The 69 participants attended a 1-year educational programme for ambulance nurses at a Swedish university.

**Ethical considerations:**

The research was conducted in accordance with the Declaration of Helsinki. Participants gave voluntary informed consent for this study.

**Findings:**

The students encountered ethical conflicts in patient relationships when they had inadequate access to the patient’s narrative. Doubts regarding patient autonomy were due to uncertainty regarding the patient’s decision-making ability, which forced students to handle patient autonomy. Conflicting assessments of the patient’s best interest added to the conflicts and also meant a disruption in patient focus. The absence of trustful relationships reinforced the ethical conflicts, together with an inadequacy in meeting different needs, which limited the possibility of providing proper care.

**Discussion:**

Contextual circumstances add complexity to ethical conflicts regarding patient autonomy, dependency and the patient’s best interest. Students felt they were fluctuating between paternalism and letting the patient choose, and were challenged by considerations regarding the patient’s communication and decision-making ability, the views of third parties, and the need for prioritisation.

**Conclusion:**

The essence of the patient relationship is a struggle to preserve autonomy while focusing on the patient’s best interest. Hence, there is a need for education and training that promotes ethical knowledge and ethical reflection focusing on the core nursing and caring values of trust and autonomy, particularly in situations that affect the patient’s decision-making ability.

## Introduction

Although different concepts are used to describe ethically problematic situations, the core meaning of this concept entails a situation in which values, norms or principles are threatened or in conflict, and a decision has to be made on how to act.^1^ Hence, ethical problems involve conflicts about the right thing to do^2^ based on an ethical conflict, that is, a conflict between legitimate values or norms.^3^ This means that ethical problems and conflicts are interwoven, meaning that ethical problems pose threats to ethical values while ethical conflicts entail a value conflict.

Working in the ambulance service involves dealing with multiple ethical problems. Students in this setting must be clinically trained to handle ethically problematic situations with multi-dimensional suffering, requiring the ability to provide medical care while creating a trustful relationship. Ambulance nurses have to make complex assessments and decisions, and take control of a situation guided by a set of conditions.^4,5^ They must also acknowledge the relationship with the patient as an important, fundamental and ethical aspect of nursing, which entails an interpersonal process in which the nurse and the patient interact.^6^ Thus, nurses have to adapt to both the patient and the environment in a dynamic relationship in order to alleviate the patient’s suffering. Such a caring relationship is related to the nurse’s commitment to providing professional care with a sufficient level of knowledge and expertise to ensure adequate care according to the patient’s needs.^7^

Studies on patients cared for by the ambulance service describe the relationship with ambulance personnel as being dependent on patients having their autonomy decreased^8,9^ and/or feeling worthless, insulted and powerless.^10^ Research from the personnel’s perspective shows different value priorities between justice, utility and rights, which could influence the relationship.^11,12^ Ethically problematic care situations involve areas such as termination of resuscitation and care in the end of life phase,^13–15^ triage,^16^ child abuse,^17^ refusal of treatment,^18,19^ delay or denial of transport for non-emergent conditions,^17^ the patient’s decision-making capacity,^16^ and the patient’s self-determination.^3^

Research has pointed to the patient relationship as the epicentre of ethical conflicts in the ambulance service, for example, regarding patient self-determination and acting in the patient’s best interest.^3^ Thus, ethical conflicts in the ambulance service may be largely related to patient relationships. In addition, compassion, objectivity and patient advocacy have been identified as key ethical values in the ambulance service.^20^ Together, this provides an understanding of the patient relationship as lying at the core of ethical deliberation in the ambulance service. In meaningful patient relationships, the nurse is described as someone upon whom the patient relies, resulting in potentially life-changing moments.^21^ In addition, getting to know patients and meeting their needs is described as being a foundation for ethically good nurse–patient relationships.^22^ Ethically problematic conditions in an ambulance care setting could be affected by the limited amount of time available to get to know the patient, no prior clinical or personal knowledge of the patient, stress, as well as the team partner.^23^ This is consistent with patients’ complaints about the ambulance service, which primarily indicate that such complaints relate to the ambulance service personnel’s rude behaviour, resulting in the patients feeling that their suffering is not being acknowledged,^24^ that they are objectified and not treated as human beings, thereby reacting negatively to the care provided.^25^ On the contrary, patients were also found to place their trust in ambulance service personnel,^26^ feeling acknowledged and treated in a dignified manner,^8^ as well as being supported after life-threatening events.^27^

Ethical problems experienced by students in clinical practice have been found to relate to the gap between moral awareness and the ideal course of action^28^ and is disrespectful of patient autonomy and dignity.^29^ The student–patient relationship from the students’ perspective is experienced to increase their own maternity and developing personal and professional values. This relationship has been found to be mechanistic (focusing on learning outcomes), authoritative (focusing on the patient’s best interest) or facilitating (focusing on the common good).^30^ Students view the relationship as more authoritative, while patients view the relationship more mechanistic.^31^ However, handling ethical conflicts in patient relationships is an important aspect of nurse education as students may lack the confidence to take an ethical stand and feel insecure about their obligation to make themselves heard in an experienced professional team.^32^ Handling ethically problematic situations as an ambulance nursing student is challenging, as they are new to the profession, lack experience and sometimes lack the knowledge to challenge older colleagues and preceptors. However, these studies mainly refer to bachelor students with no previous nursing experience.^28,29,32^ Thus, the ethical conflicts encountered by specialist ambulance nursing students may vary.

In conclusion, patient relationships in the ambulance service are challenging. This emphasises the importance of the ability of ambulance nursing students to identify ethical conflicts in the patient relationship in order to avoid extended suffering, which poses challenges for student education. However, the knowledge of ethical conflicts in student–patient relationships is limited. To our knowledge, there is no previous study that describes ethical conflicts experienced by students in the ambulance service, either from ambulance nursing students’ perspective or from paramedic students’ perspective. Thus, the aim of this study was to describe ethical conflicts in patient relationships as experienced by ambulance nursing students during clinical studies.

## Research design

The study design was exploratory and interpretative in order to inductively analyse textual data from two examinations in clinical placement courses for ambulance nursing students (henceforth referred to as ‘students’).

### Participants and research context

Ambulance nurse education and training in Sweden comprises a 1-year master’s degree and a postgraduate diploma in specialist nursing for registered nurses (RNs). In order to qualify for the programme, students must be registered as an RN with a Bachelor of Science degree, including specialisation in caring or nursing science. The master’s degree is conducted in accordance with course requirements of 60 European Credit Transfer System (ECTS) credits including at least 30 credits relating to advanced studies in caring science.^33^ The learning process for becoming a specialist nurse in ambulance care comprises both theoretical knowledge and clinical training, which is mainly carried out in the ambulance service. The participating students attended the Postgraduate Diploma in Specialist Nursing – Prehospital Emergency Care programme.

The programme was conducted at a university in Western Sweden, in a region comprising 23,797 km^2^ and 1.7 million inhabitants. A total of 70 students were asked to participate and 1 student declined. Thus, 69 students agreed to participate, comprising both women (n = 27, 39%) and men (n = 42, 61%) (Table 1).

**Table 1.** Participant demographics.

	n	%	Median (range)	Mean
Sex^a^				
Female	27	39		
Male	42	61		
Age,^b^ years			33 (25–50)	34
Workplace as RN^b^				
Ambulance care	40	71		
Emergency care (in-hospital)	11	20		
Surgery	1	2		
Medicine	1	2		
Other/unknown	3	5		
Experience of ambulance care,^c^ years			5 (0–18)	6

RN: registered nurse.

^a^ n = 69.

^b^ n = 56.

^c^ n = 40 (those with workplace ‘Ambulance care’).

During the programme, students attended 10 weeks of full-time clinical studies in ambulance service organisations in Southern Sweden. All organisations comprised a system of Advanced Life Support (ALS) units with at least one RN in each ALS ambulance, usually a specialist RN in ambulance care or in another specialist nursing area. The proportion of RNs in the ambulance service who are specialist ambulance nurses varies considerably across organisations and represents 20%–80% of the nursing staff. The proportion of paramedics/emergency medical technicians varies between 15% and 35% of the total ambulance service staff. Thus, the dyadic ALS team comprises different professions and education levels, requiring the ability to co-operate and provide advanced assessments and care. Emergency physicians who are clinically active in the Swedish ambulance service are extremely rare. Instead, they have an administrative responsibility for medical issues and staff training.^34^

### Data collection

Data were collected in three different student groups on the clinical caring science course ‘Prehospital emergency care’ between 2014 and 2016. In a written examination, students were encouraged to describe an ethically problematic patient relationship. This resulted in a total of approximately 160 pages (A4) of single space text. The aim of the written examination was that the students, based on their own experiences, should initially identify and problematize ethical problems and conflicts in a specific patient relationship that they encountered during their clinical studies. Second, the students should suggest potential solutions to the ethical conflicts they had described. In this study, only the descriptions of the ethical conflicts have been analysed, not the way in which the students handled the conflicts.

### Data analysis

The data were analysed using thematic analysis,^35^ a flexible method in several qualitative studies. In this study, the method is used exploratively, that is, data are analysed without a pre-defined theoretical basis.

The analysis was conducted in six stages^35^: (1) The examinations were read and re-read several times in order to gain familiarity with the data as a whole; (2) the examinations were equally distributed between both authors who individually extracted and coded meaning-units related to ethical conflicts in patient relationships; (3) the codes were critically discussed by the authors until consensus on internally coherent, consistent and distinctive codes was reached and themes were generated using codes relevant to each other; (4) the themes were critically reflected upon together with the extracted codes in each examination and the data corpus, until a thematic map of ethical conflicts in patient relationships emerged; and (5) themes were defined, the attributes of each theme were clarified, and a clear definition and name for the theme was presented. These texts and maps are collectively referred to as a whole text, and a comprehensive map was produced for all texts. The themes were re-processed, re-defined and re-clarified in order to obtain fewer distinct themes. The last step (6) was to present the themes and a coherent pattern.

The authors are specialist ambulance nurses with clinical experience from ambulance care and working as senior lecturers. The authors’ pre-understanding was continuously questioned, discussed and reflected upon throughout the research process in order to promote credibility and validity.

### Ethical considerations

According to Swedish law, this study does not need to be reviewed by the Swedish Ethical Review Authority.^36^ However, the study underwent ethical discussions beforehand at the university and is in accordance with the Declaration of Helsinki.^37^ Prior to data collection, the participants received both written and verbal information. They were informed that they could withdraw from the study at any time without stating a reason and that participation was voluntary. All participants signed an informed consent form.

## Findings

The experiences of ambulance nursing students regarding ethical conflicts in patient relationships emerged as six main themes comprising 19 sub-themes (Table 2).

**Table 2.** Main themes and sub-themes.

	Sub-themes	Main themes
1	A deficient conversation	Inadequate access to patient narratives
	The patient’s limited ability to communicate	
	Lost communication	
2	Unsure about the patient’s decision-making ability	Uncertainty about patient autonomy
	Wants to disregard the patient’s autonomy	
	Obligated to disregard the patient’s autonomy	
3	A lack of support in the student’s own assessment	Conflicting assessments of the patient’s best interest
	Decisions are questioned by significant others	
	Disagreement between patient and significant others	
4	Significant others deprive the patient of their autonomy	Disruption to patient focus
	Significant others are acting as the patient’s surrogate	
	Significant others are making the patient insecure	
	Bystanders ruin patient relationships	
5	Patient refusal to co-operate	Absence of trustful relationships
	Lost reciprocal trust	
	Influenced by prejudices and aversions	
6	Powerless while confronting the patient’s suffering	Limited opportunity to provide proper care
	Inadequacy in accommodating the needs of significant others	
	Forced to prioritise between the patient’s needs	

### Inadequate access to patient narratives

There are several reasons why adequate access to the patient’s narrative is not possible. The patient’s condition could range from being unconscious to wanting, but being unable, to communicate properly. This means that there is no basis for shared decision-making, resulting in ethical conflicts related to the patient’s inability to give their consent.

The absence of a good care relationship is indicated when there is *a deficient conversation* with the patient. This deficiency arises when the patient has difficulty explaining their situation, is confined, only speaks a few words or whose body language is inconsistent with their verbal communication. A deficiency arises when it is impossible to get sensible or clear answers from the patient. Instead, the answers are perceived as unclear, inconsistent or vague. Also, a deficiency arises when the patient’s language is inadequate or the patient refuses to co-operate. Despite their best efforts, it is impossible to understand the patient’s problem, risking delayed care and limited participation.

*The patient’s limited ability to communicate* is a source of ethical problems. Situations that deal with patients who have not mastered the Swedish language can cause such ethical problems, but these problems can also arise when conversations are conducted via an interpreter. The inability to speak Swedish leads to misunderstandings, mistakes and frustration. The use of interpreters restricts patient participation and makes it difficult to ask sensitive questions, which causes insecurity and makes it difficult to provide comfort to the patient.

Ethical conflicts arise when no dialogue can be conducted with the patient, resulting in *lost communication*. Sometimes it is not possible to communicate with the patient or family members. Instead, the relationship is based on interpretations of the patient’s situation. This lack of communication and consequent access to the patient’s narrative takes place with patients who are unreceptive to conversations or who do not answer questions. Occasionally, establishing a dialogue with a patient is completely impossible.

### Uncertainty about patient autonomy

Taking patient autonomy into account is ethically problematic in various ways. While training in patient assessment, it is difficult to determine whether patients understand their situation, the information provided and the available care options. The problem is also about depriving patients of their autonomy despite the patients being regarded as competent.

The students are *unsure about the patient’s decision-making ability* because of their medical condition, which is considered to affect the patient’s ability to respond to questions and make it difficult to assess how much the patient understands. This concerns patients who are not fully orientated or who are unconscious, for example, when patients have no insight into their disease or are under the influence of drugs. The ethical conflicts derive from uncertainty about the patient’s decision-making ability. Thus, students are forced to decide whether or not the patient is able to make autonomous decisions.

Ethical conflicts arise when the student *wants to disregard the patient’s autonomy*. This involves situations in which the patient must be persuaded to follow the student’s recommendation in order to receive proper care. Other problems arise when students believe they know better than patients about their situation. Doubt arises based on a concern that the desire to disregard patient autonomy is ethically problematic and very often questionable.

Sometimes students feel *obligated to disregard the patient’s autonomy*, for example, when the patient’s condition is life threatening or when preceptors are unable to preserve patient autonomy. Using force in order to provide care is regarded as necessary in certain situations but also constitutes an ethical problem. For example, this occurs in situations in which patients show an inability to defend their autonomy. However, ethical conflicts arise from the students’ uncertainty as to whether providing care is in accordance with the patient’s preferences and wishes.

### Conflicting assessments of the patient’s best interest

Ethical conflicts emerge from conflicting assessments of the patient’s best interest. A risk of decreasing patients’ trust occurs when the preceptor overrules the student. Competing views of significant others and the patient make it difficult to make decisions based on the patient’s best interest.

*A lack of support in the student’s own assessment* means being questioned by the patient, preceptors, other healthcare professionals or significant others. This manifests as the students being deprived of their own influence over the patient relationship. For example, having ideas about how to provide care and then being overruled decreases experience-based learning outcomes. In some situations, the assessment and care of the patient ends up being the responsibility of different physicians, leaving the student irresolute and in conflict with the patient’s best interest.

Students occasionally feel that their *decisions are questioned by significant others*. This is an ethical problem when it limits the ambition of properly training students in assessments and decision-making in line with the most beneficial measures for meeting the patient’s needs. Sometimes, significant others even decline hospital transport, thereby violating patient safety and putting the student in a precarious position.

Ethical conflicts become apparent when the patient and significant others have different opinions and a *disagreement between patient and significant others* occurs. This is problematic when the patient is adapting to the wishes of significant others, even if it is not congruent with the wishes of the patient. It is also problematic when the patient’s wishes or preferences are deemed to jeopardise their safety and are in conflict with the shared opinion of the student, preceptor and significant others, violating the patient relationship.

### Disruption to patient focus

Disruption to patient focus emerges as an ethical problem when the student is unable to put the patient at the centre of the relationship. This disruption relates to interaction with others and the impact on the relationship. Other people acting on behalf of the patient are perceived as decreasing the patient’s ability to maintain their autonomy, disrupting the students’ patient focus while they are learning to establish a good patient relationship.

Ethical conflicts arise when *significant others deprive the patient of their autonomy*, act on behalf of the patient and incapacitate the patient, even in situations in which patients express the desire to not have significant others present, and this is not respected. Ethical problems also arise out of the students’ uncertainty as to whether or not the significant others are capable of making decisions on the patient’s behalf.

In situations in which the *significant others are acting as the patient’s surrogate*, the patient’s focus is disrupted. This involves, for example, situations in which the students ask the patient questions but receive answers from the significant others. The focus then shifts from the patient to the significant others. In addition, when the suffering of the significant others is recognised, this fragments the students’ focus on the patient relationship, causing frustration.

Occasionally, the students feel that the *significant others are making the patient insecure*. This is described as being ethically problematic as the significant others are contributing to the patient’s suffering, for example, in situations involving the patient’s fear of significant others who are aggressive or when parents transfer their concerns to their sick child.

Ethical problems are further described when *bystanders ruin patient relationships*, for example, when a trustful relationship with the patient, established by the student, is ruined by the irritation, disrespect and disinterest of ED personnel. This is also felt when preceptors who do not share the students’ ethical values leave the students in doubt.

### Absence of trustful relationships

Ethical problems arise as a consequence of failing to establish a trustful relationship with the patient. There are three reasons for this: First, trust means trusting each other but occasionally *reciprocal trust is missing.* The second reason is when *the patient refuses to co-operate.* Third, trust cannot be established when the relationship is *influenced by prejudices and aversions*.

Sometimes a *patient will refuse to co-operate*, meaning it becomes impossible to establish trust when the patient refuses to answer questions, is unwilling to talk or reacts to being touched or examined. The absence of trust also has its foundation in aggressive, threatening and unreasonable patients. In such situations, it is difficult to get close to the patient, risking further aggression. Instead of confidence-building measures, caution and control are prioritised.

When students experience *lost reciprocal trust*, there is no real basis for a genuine relationship; it is difficult to create a personal encounter and to be honest. For example, mutual trust is threatened by preceptors questioning the patient, when it is impossible to rely on the patient and when the patient is in a state of panic.

The relationship is occasionally *influenced by prejudices and aversions*. The preceptor’s or the student’s own presumptions risk adversely affecting their relationship with patients and family members. The attitudes and values of preceptors occasionally mean that the relationship has been pre-defined in a judgmental and prejudicial manner, for example, by stating that the patient is behaving in a threatening manner.

### Limited opportunity to provide proper care

Being forced into making priorities when feeling powerless and insecure is described as ethically problematic and a limitation to providing proper care. Problems arise when students wonder if the care they are providing could have been done differently or whether they have interpreted the patient’s situation correctly, prioritising between the patient’s needs. The students have an idea of what proper care means but regard their limited ability to provide this as being an ethical problem.

Being *powerless while confronting the patient’s suffering* means that the students feel moral distress. This includes situations in which students see that a patient is suffering but are unable to alleviate their suffering, for example, when being forced to accept a low prioritisation of the patient in the ED, even if the students have made a higher priority.

Limited opportunities are ethically problematic when students feel a sense of *inadequacy in accommodating the needs of significant others*. The students feel that they are completely occupied with patient care and not being able to focus on significant others who need care. This is described in situations in which CPR (cardiopulmonary resuscitation) leaves significant others alone in a traumatic life event, or when students are unable to focus on the children of critically ill parents. Ethical problems also relate to feeling insecure regarding how to assess and respond to social misconduct in the patient’s family situation.

Students feel that they are being *forced to prioritise between different needs*, as patients have multiple needs simultaneously. Thus, students feel that they do not know what is ethically good for the patient, for example, deciding in a CPR situation whether or not resuscitation should continue. Patients’ needs are then not addressed and are fragmented in prioritising between the needs of patients, significant others and their own needs. Sometimes the student’s own needs are considered to be more important than the needs of the patient, for example, in situations in which the student’s personal safety is prioritised.

## Discussion

These findings give an insight into the ethical conflicts in patient relationships faced by ambulance nursing students. Contextual circumstances appear to add even more complexity to the perceived conflicts when considering patient autonomy, dependency and the patient’s best interest. The students want to both disregard patient autonomy and are occasionally obligated to disregard patient autonomy and change to a paternalistic stance. The students feel they are fluctuating between paternalism and letting the patients make their own decisions, wondering how to help the patients and meet their needs. At the same time, students are challenged by considerations regarding the patient’s communication and decision-making ability, views of third parties, and the need for prioritisation.

Here we will focus on the students’ experiences in relation to three components of ethical competence in nursing, identified by Lechasseur et al.^38^ as *ethical sensitivity, ethical knowledge* and *ethical reflection*. The students’ handling of ethical conflicts will be discussed elsewhere, in relation to three other components of ethical competence: ethical decision-making, ethical action and ethical behaviour.^38^

### Patients’ decision-making ability and the ethical basis of autonomy

Access to patient narratives is an important aspect of the relationship in order to understand the perceived illness and suffering as a human experience rather than a biomedical phenomenon.^6^ Hence, when access is limited, it appears to trigger the students’ ethical sensitivity and direct it towards the patient’s impaired decision-making ability, or inability to express their own wishes. The lack of communication compels the students, when acting as clinicians, to assess and make decisions based on their own perception of what is good for the patient. This creates a sense of uncertainty regarding their own decisions in relation to decisions that would have been made if the patients had been able to participate. The students recognise this potential source of ethical problems. Ethical sensitivity has previously been studied among nursing students,^39^ showing the third highest ranked moral issue was the responsibility to know the patient’s situation. Unfortunately, as our findings indicate, when the patient’s narrative cannot be obtained, it is more difficult to understand the patient’s situation. However, being aware of this responsibility does not necessarily mean that students have the ability to act in a way that feels safe, reduces uncertainty and is considered ethically justified.

The findings show that the patient’s decision-making ability is a matter of the degree of ability. Based on Sandman and Munthe’s^40^ definition of autonomy as related to the person’s decision-making and acting according to their own preferences, which they control, the students’ insecurity regarding the patient’s decision-making ability makes them uncertain as to how to manage patient autonomy. Importantly, students do not appear to fully understand the potential role of promoting patient autonomy through their relationship. This is interesting in light of relational autonomy suggesting that as well as ensuring the patients’ informed consent, healthcare professionals are important in promoting patient autonomy^41^ and are therefore alert to conditions that affect the patients’ capacity for autonomous reasoning. Following this, respect for autonomy and for patients whose autonomy is impaired entails an obligation on the part of healthcare professionals such as ambulance nurses.^42^ Thus, the students’ theoretical ethical knowledge and ability to ethically reflect on these alternative ways of thinking about autonomy are lacking. This corresponds to nursing students as becoming professionals, still lacking the confidence to take an ethical stand and confront the ‘real world’ of healthcare.^32^ At the same time, the patient relationship is experienced to develop students’ personal and professional values, primarily with the student focusing on the patient’s best interest.^30^ However, for ambulance nursing students, this may involve an encounter with a previously unfamiliar caring environment that may reveal new ethical issues in patient relationships.

The students do not fully understand the ethical value of autonomy per se or when autonomy should be considered an intrinsic value for achieving other important values. As a result, their wish to disregard the patient’s autonomy does not necessarily mean that it is about ignoring patient autonomy, and when students feel obligated to disregard patient autonomy, it is usually in situations in which no other ethically justifiable option is available. Hence, ethical problems arise in the tension between the patient’s decision-making ability and the student’s notion of the ethical basis of autonomy. In a study by Erdil and Korkmaz,^43^ respect for patient autonomy was frequently violated, as witnessed by nursing students in clinical studies. In this regard, ethical decision-making models have previously been found to be helpful among nursing students in encountering ethically challenging situations.^2^ Thus, the present findings are relevant in terms of different models of shared decision-making, models that can be considered to be versions of paternalism or patient choice. However, the meaning of autonomy is complex and may be valued as a property of a person’s life rather than a property of the person.^40^ In this regard, the concept of patient advocacy is well established in nursing but lacks research from a paramedic perspective.^44^ Thus, the present study provides unique understanding of ethical dimensions of the nurse–patient relationships in the ambulance service context. A tentative interpretation of our findings, based on shared decision-making, is that students cannot use the ideal model, that is, based on ‘shared rational deliberative joint decisions’, but instead are (subconsciously) forced to use less ideal models. In order to address the complexities, Sandman and Munthe suggest that the healthcare professional and the patient engage in a shared rational deliberation in which the patient ultimately decides or, alternatively, the patient and the professional engage in a rational deliberation that is brought to a consensus and results in a joint decision.

### Lost patient focus and the influence of others

The students show ethical sensitivity and face problems when other persons have contrary views to their own. However, students fully understand how different views can be balanced and communicated, and which alternatives should be proposed based on ethical reflection and analysis.

Conflicts arise when the student faces opposition from others after having proposed an action plan. The questioning of the student’s assessment of what is best for the patient means that new alternative solutions must be sought, which leads to a temporary paralysis of action. Previous research^2,45^ shows that nursing students encounter ethical problems in relation to RNs and other healthcare professionals who are perceived to provide poor care or risk harming patients. In our findings, the students perceive that they themselves have the right/good solution and give examples of wrongful and unethical actions on the part of their preceptors or their colleagues. Some of these descriptions appear to be well founded although it is not possible to assess the accuracy of the students’ statements. This is in line with Axelsson et al.,^46^ who found that students’ experiences in clinical ambulance studies seem to be affected by the preceptor and how he or she interacted in relation to the student and the patient.

Students are also sensitive to disturbances in their patient focus, which may threaten the patients’ trust in the students. This means that the patient risks being harmed or is harmed as a consequence of a lack of care or improper care and also that the student’s confidence-building work vis-à-vis the patient is made difficult or fails. The students show relational knowledge based on normative thoughts about the patient’s best interest, but above all they try to apply ethical reflection ‘in action’ on the probable consequences for the patient. Hence, based on the student’s perspectives and values, ethical problems arise when the patient, family members or preceptors oppose the appropriate action plan that the student intends to use in order to alleviate the patient’s suffering.

### Absence of trust and the need to prioritise

The findings show that there is no firm basis for a relationship when trust is absent. Trust is described as being a core of the patient relationship and the starting point for the nurses’ responsibility,^47^ and the most important component of medical students’ perceptions of professionalism.^48^ In some situations, patients may trust out of necessity rather than willingness while suffering from perceived acute illness/injury.^49^ However, the students do not appear to understand the asymmetric responsibility in the relationship, that is, that this is primarily their responsibility.^47^ Thus, it is important to understand what patients risk in the relationship, such as an imbalance in power in the nurse–patient relationship putting the patient in a vulnerable position.^50^ On one hand, the students are sensitive and discover the point when the trust between themselves and the patient fails. On the other hand, they seem quite unaware that they themselves may have caused the loss of patient trust. Instead, responsibility for the loss of trust is placed either on the patient or occasionally on both student and patient.

Students feel restricted in providing proper care and sometimes also powerless when confronting the patient’s suffering. When the patient is exposed to an obvious life threat, it is relatively easy to understand what must be prioritised. This is in line with previous findings that describe the dichotomous approach of ambulance nurses to medical care and caring relationships as being an obstacle to a holistic approach.^51^ This could lead to unethical consequences while excluding important aspects of patients’ suffering or when there is an obligation to prioritise between different patient needs, and students sometimes prioritise these needs in relation to family needs.^3,13,14^ Faced with these choices, the students do not fully understand that alternative pathways should be tried or that their sense of inadequacy must sometimes be dealt with on the basis of reflection ‘in action’ in order find a reasonable solution.^52^ Being an ambulance service professional has previously been described as being an authority in patient relationships,^53^ engendering an explicit moral course of action. Many of the students in this study have previous experience of ambulance care. However, training to become a specialist nurse requires the application of theoretical caring science-based knowledge to clinical care training at an advanced level. This could be a problem if a knowledge gap exists between the students’ theoretical knowledge and the moral course of the clinical care.^28^ Hence, ethical conflicts arise when students fail to create trust in the relationship, which also complicates the prioritisation between needs and the ability to provide proper care.

### Methodological considerations

The participants constitute a representative group of students based on the authors’ extensive experience in training ambulance nursing students. However, it is possible to question whether the participants represent the student perspective only or whether the results also apply to experienced nurses in the ambulance service, since the participants have a significant variety in the number of years of working in this setting. This need not necessarily constitute a limitation but rather a strengthening of the transferability of our findings to other pre-hospital care contexts.

The analysis was conducted in co-operation between both authors and discussed until consensus was reached according to the sub-themes and themes. This mode of analysis strengthens the validity and reliability because of the researchers’ approach to investigating, checking and questioning the data, coding and themes.^54^ However, there is a risk that the authors’ pre-understanding and presumptions may have negatively impacted the results. This risk has been partially reduced by the authors’ questioning the presumptions throughout the research process. In addition, one of the authors validated the findings by means of seminars with the participants about their written examinations.

## Conclusion

This study provides unique knowledge of ambulance nursing students’ experiences regarding ethical conflicts in patient relationships during clinical studies. This knowledge relates to the level, attitude and approach in the student–patient interaction within the specific context of ambulance care. When the patient is unable to fully respond and interact, questions arise about the patient’s decision-making ability. The lack of ability challenges the student’s own ability to manage and promote patient autonomy, as well as handle the views of third parties. In addition to the ethical conflicts, the nature of the situation usually requires a need for prioritisation. Consequently, the essence of the patient relationship appears to be a struggle to preserve autonomy while focusing on the patient’s best interest. Regarding the students’ ethical competence, comprising ethical sensitivity, ethical knowledge and ethical reflection, it appears to be particularly important that specialist education and training in ambulance care promote ethical knowledge and ethical reflection that focus on the core nursing and caring values of trust and autonomy, especially in situations that affect the patient’s decision-making ability.
